# Lag effect of climatic variables on dengue burden in India

**DOI:** 10.1017/S0950268819000608

**Published:** 2019-04-03

**Authors:** Satya Ganesh Kakarla, Cyril Caminade, Srinivasa Rao Mutheneni, Andrew P Morse, Suryanaryana Murty Upadhyayula, Madhusudhan Rao Kadiri, Sriram Kumaraswamy

**Affiliations:** 1 Applied Biology Division, CSIR-Indian Institute of Chemical Technology, Tarnaka, Hyderabad-500 007, Telangana, India; 2 NIHR Health Protection Research Unit in Emerging and Zoonotic Infections, Liverpool L69 3GL, UK; 3 Department of Epidemiology and Population Health, Institute of Infection and Global Health, University of Liverpool, Liverpool L35RF, UK; 4 Department of Geography and Planning, School of Environmental Sciences, University of Liverpool, Liverpool L69 7ZT, UK; 5 National Institute of Pharmaceutical Education and Research, Guwahati-781 032, Assam, India

**Keywords:** Dengue, distributed lag non-linear model, El Niño, India, relative risk, temperature

## Abstract

Dengue is a widespread vector-borne disease believed to affect between 100 and 390 million people every year. The interaction between vector, host and pathogen is influenced by various climatic factors and the relationship between dengue and climatic conditions has been poorly explored in India. This study explores the relationship between El Niño Southern Oscillation (ENSO), the Indian Ocean Dipole (IOD) and dengue cases in India. Additionally, distributed lag non-linear model was used to assess the delayed effects of climatic factors on dengue cases. The weekly dengue cases reported by the Integrated Disease Surveillance Program (IDSP) over India during the period 2010–2017 were analysed. The study shows that dengue cases usually follow a seasonal pattern, with most cases reported in August and September. Both temperature and rainfall were positively associated with the number of dengue cases. The precipitation shows the higher transmission risk of dengue was observed between 8 and 15 weeks of lag. The highest relative risk (RR) of dengue was observed at 60 mm rainfall with a 12-week lag period when compared with 40 and 80 mm rainfall. The RR of dengue tends to increase with increasing mean temperature above 24 °C. The largest transmission risk of dengue was observed at 30 °C with a 0–3 weeks of lag. Similarly, the transmission risk increases more than twofold when the minimum temperature reaches 26 °C with a 2-week lag period. The dengue cases and El Niño were positively correlated with a 3–6 months lag period. The significant correlation observed between the IOD and dengue cases was shown for a 0–2 months lag period.

## Introduction

Dengue is one of the most important mosquito-borne viral diseases in tropical and sub-tropical countries. It is transmitted through the bite of *Aedes* mosquitoes infected with dengue virus (DENV 1–4 serotypes). *Aedes aegypti* and *Aedes albopictus* are believed to be the main vectors of dengue virus in India [1, 2]. Dengue causes a wide range of clinical symptoms including asymptomatic cases, acute febrile syndrome, severe and fatal cases of haemorrhagic manifestation that result in a significant fluid loss, which ultimately leads to shock. During the past five decades, the incidence of dengue has increased 30-fold and it has become a major public health problem globally [3]. In 2012, WHO estimated that 50–100 million new dengue infections were occurring annually. Before 1970, only nine countries had experienced severe dengue epidemics. The disease is now endemic in more than hundred countries across the world. Over 50% of the world's population, mostly in the tropics, is identified at risk of dengue infection [4].

Dengue is highly endemic in Southeast Asia and the Western Pacific regions [5]. In recent years, the number of dengue cases also sharply increased in Southern, Northern and Central Americas [6]. Cases across the Americas, Southeast Asia and the Western Pacific have exceeded 1.2 million in 2008, 2.2 million in 2010 and over 3.2 million in 2015 [7]. In Southeast Asia, the disease has been one of the major causes of hospitalisation amongst children since the 1990s [8]. Asian countries contribute around 70% of global dengue burden. India alone contributes about 34% global dengue burden and two-thirds of India's population are estimated to be at risk of dengue infection [5].

Since the mid-1990s, dengue epidemics in India have become more frequent in urban zones and they also spread to new geographic regions [9]. This geographic expansion of dengue might be related to changes in eco-climatic factors, climate change, rapid urbanisation, rapid population growth, population movement and ineffective vector control operations [10]. The epidemiology of dengue in India was first described in 1780 and the first large-scale outbreak occurred in 1963 [11]. Later, subsequent outbreaks have been reported from different parts of India [2, 11]. India has reported all four serotypes (DENV 1, 2, 3 and 4) of dengue virus since 1956 for various parts of the country [12]. Since 2001, the total number of dengue cases has steadily increased in India. In the early 2000s, dengue was endemic in a few southern (Maharashtra, Karnataka, Tamil Nadu and Pondicherry), northern and northwestern states (Delhi, Rajasthan, Haryana, Punjab and Chandigarh). Recently, dengue expanded to many states including the Union Territories [9]. Not only has the number of cases and severity of disease also increased, dengue used to be restricted to urban areas, but it has now spread to rural areas [1].

Like most vector-borne diseases (VBD), the epidemiology of dengue consists of host (humans), vectors (*Aedes* mosquitoes) and pathogen (dengue virus). The VBD system is strongly influenced by climatic factors including temperature and rainfall. The global temperature has increased significantly over the 20^th^ century. By the end of the 21^st^ century, it has been predicted that the global mean temperature will rise between 1.1 and 6.4 °C with respect to pre-industrial values [13]. Overall, these rising temperatures will enhance the transmission rate of mosquito-borne diseases and will allow the expansion of the vector into new geographic regions [14]. According to the Intergovernmental Panel on Climate Change (IPCC), approximately 1.5–3.5 billion people worldwide will be at risk of dengue infection by 2080 due to climate change [13]. Temperature affects the life cycle of *Aedes* vectors including development and survival of the immature and mature stages, development and length of the gonotrophic cycle. Additionally, temperature also influences virus replication rates within the mosquitoes, their gonotrophic cycle and the adult size of mosquitoes [15]. High-temperature conditions tend to shorten the extrinsic incubation period within the mosquito vector and thus increase the odds of more mosquitoes becoming infectious during their life span. Rainfall provides vector breeding habitats, although the relationship between rainfall and dengue is non-linear; heavy rainfall can flush out breeding sites as well [16]. Relative humidity also favours the survival rate and biting activity of adult mosquito. This allows the infected female mosquitoes to complete more than one replication cycle of the virus [17].

The El Niño Southern Oscillation (ENSO) is a naturally occurring mode of tropical Pacific climate variability and it has a large impact on global and regional temperature and rainfall in the Tropics [18]. El Niño events are characterised by an increase in sea surface temperatures (SST) in the tropical eastern Pacific Ocean, while La Niña events are characterised by cooler than average SST in the same region. These events typically occur every 2–7 years and develop in association with large-scale oscillations in atmospheric pressure over the tropical Indian and Pacific oceans [19]. Studies have shown that there is an association between ENSO and the burden of mosquito-borne diseases in tropical and subtropical regions [20–22]. ENSO has a significant impact on the Indian monsoon and influences weather patterns worldwide [23]. El Niño events are associated with drier than average conditions in the Indian region [24]. Conversely, La Niña events are associated with increased rainfall in India. El Niño events have been positively associated with an increase in the number of malaria cases in Venezuela and India [25, 26]. However, a weak association was observed between ENSO events and dengue incidence in Bangladesh [27].

The Indian Ocean Dipole (IOD), also called the Indian Ocean Zonal Mode, was discovered in the late 1990s. The IOD is a natural climate mode of variability; it arises from ocean–atmosphere interaction, and is the largest mode of interannual climate variability in the tropical Indian Ocean [28, 29]. The IOD mode is characterised by the anomalous west–east SST gradient accompanying zonal wind anomalies over the equatorial Indian Ocean [28]. The positive phase of the IOD is associated with warmer than average SST in the western Indian Ocean; colder than average SST conditions in the southeast Indian Ocean, off of Sumatra; and anomalous easterlies appear around the central equatorial Indian Ocean [28]. During a positive IOD event, the East African region receives above normal rainfall, while rainfall is reduced in Indonesia and in Australia causing significant drought [30]. The IOD also plays an important role as a modulator of the Indian summer monsoon rainfall (ISMR) and influences the correlation between the ISMR and ENSO [31].

There are only a few studies that investigate the epidemiology of dengue virus and the impact of climate on dengue transmission across India [16, 32]. Researchers have provided various predictions at global scale based on climate models. Hence, the current study aimed to estimate the effect of climate on dengue transmission in overdispersed datasets. A distributed lag non-linear model (DLNM) was used to examine the delayed lag effect of different climatic variables on dengue cases using data from 2010 to 2017 [33]. DLNM is a flexible model which simultaneously describes a non-linear and delayed effect of different factors on disease burden of climate change on dengue incidence [34]. We utilised the DLNM method to investigate the lag effects of climate variables on dengue cases in India. This technique is based on the cross-basis function that examines a two-dimensional relationship along the dimensions of climate change (rainfall or temperature) and time lag, in weeks [33]. In addition, the best-fitting model with climate parameters will be used to develop a dengue forecast model for India. The results of the study may also provide useful information to allocate public health resources and mitigate the burden of dengue disease in India.

## Methods

### Epidemiological data

Weekly reports of dengue cases for India from 2010 to 2017 were collected and compiled by the Integrated Disease Surveillance Program (IDSP), Ministry of Health and Family Welfare, Government of India (http:\\www.idsp.nic.in). Dengue cases are confirmed in the laboratory by the MAC ELISA method on the basis of the detection of IgM antibodies [32]. The institutional committee (CSIR – Indian Institute of Chemical Technology) approved the study and no patient samples were handled during the study. Hence, the consent from patients was waived as we dealt with the recorded data.

### Climate data

**Fig. 1.**
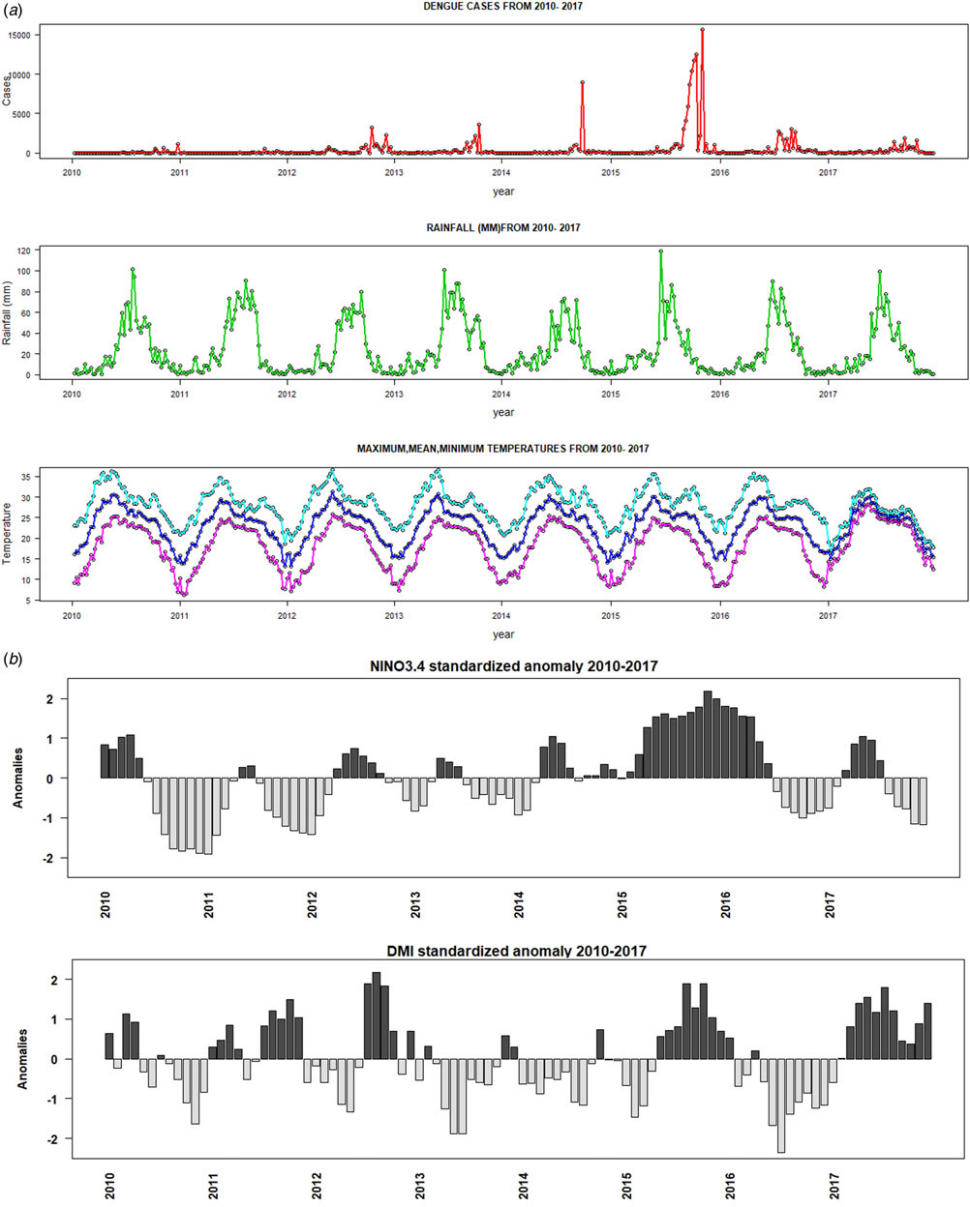
Time-series plots of (a) weekly dengue cases, precipitation, maximum, minimum and mean temperature, (b) Nino3.4 and DMI indices during the period 2010–2017.

The temperature data were derived using the NCEP-DOE 2 reanalysis dataset [35]. This reanalysis dataset is produced using a state-of-the-art analysis/forecast system to perform data assimilation using past observed climate data. The NCEP reanalysis data are a mix of climate model data, corrected by a large amount of climate observations (using assimilation techniques to produce a continuous gridded product in space and time as climate observations can be patchy). The model component of the reanalysis is consequently based on a climate model – some physical processes are resolved in the spectral grid space. This dataset is available on a T62 (spectral model grid truncation, it has pros and cons for solving physical processes in the earth system) global Gaussian grid (about 1.8° × 1.8°) from 1979 to present at daily time step. Rainfall was derived from the Tropical Rainfall Measuring Mission (TRMM) dataset. TRMM is available on a 0.25° × 0.25° spatial grid covering the Tropics and sub-Tropics (60°N-60°S) from 2010 to 2017 [36]. Time series of weekly rainfall, maximum, minimum and mean temperatures for India from 2010 to 2017 are shown in Fig. 1. Statistics summarizing the average weekly temperatures (maximum, minimum and mean temperatures) and rainfall are shown in Table 1.

**Table 1.** Descriptive statistics of weekly information on weather and dengue cases from 2010 to 2017

Descriptive statistics of weekly data							
Variable	Mean	Minimum	Maximum	SD	Percentile		
					25%	50%	75%
Cases	419.5	0	15 619	1475.54	10	56	211
Mean temperature(°C)	23.04	13.15	31.24	4.48	18.91	24.48	26.19
Maximum temperature (°C)	28.02	17.07	36.69	4.15	25.03	28.32	30.71
Minimum temperature (°C)	18.18	6.25	28.11	5.49	13.15	19.41	22.80
Rainfall (in mm)	23.22	0.11	119.09	25.01	3.65	12.98	38.82

The monthly El Niño-Southern Oscillation (ENSO) index used in this study is the Nino3.4 SST index (calculated for the region 170°E-120°W and 5°N-5°S) available from the Climate Prediction Centre of the US National Weather Service (http://www.cpc.ncep.noaa.gov/data/indices/sstoi.indices) [37]. Similarly, the IOD is represented by the SST difference between the western equatorial Indian Ocean (50°E-70°E and 10°S-10°N) and the southeastern equatorial Indian Ocean (90°E-110°E and 10°S-0°N). This SST gradient is also known as the Dipole Mode Index (DMI). When the DMI is positive, the phenomenon is referred to as the positive phase of the IOD, and when it is negative, it is referred to as the negative phase of the IOD. The monthly DMI values were obtained from the Japan Agency for Marine Earth Science and Technology (http://www.jamstec.go.jp/frcgc/research/d1/iod/DATA/dmi.monthly.txt) [28].

### Lag effects

DLNM were separately fitted to investigate if there was a delayed impact of climatic factors on weekly dengue cases at country scale. The relationship between climatic factors and dengue cases was investigated by employing two distinct approaches. First, the association between dengue and climatic factors at different time lags (lag 0–25 weeks) was assessed using Pearson correlation analysis. We correlated the value of a particular climatic variable at week *i* with the number of dengue cases by date of symptoms expressed at week *i* + *τ* where *τ* is the time lag (in weeks). The second part of the analysis was carried out using the DLNM package available in R software. We used DLNM combined with quasi Poisson regression analysis. The observed data of weekly dengue cases show overdispersion (e.g. the variance of dengue cases largely exceeds the mean), consequently quasi-Poisson regression was used to estimate the effects of independent variables (observed rainfall and temperature) on dependent variable (observed dengue cases). The median value of climatic parameters (Table 1) was defined as the baseline centring value for calculating relative risk (RR). The RR was based on the Poisson regression model adjusting for various confounders. RR is defined as ‘the ratio of the probability of dengue occurring at a certain value of a climate variable to the probability of the event occurring at a reference value of the same climate variable’.

The DLNM framework has enough flexibility to represent the non-linear and delayed associations on lag scale between climatic factors and dengue cases based on cross basis function. Lag represents the time interval between exposure event and clinical outcome.
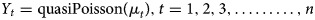

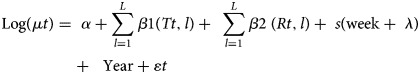
where *t* is the week of observation, *Y*_*t*_ denotes the observed dengue counts in week *t*, log(*μt*) represents the logarithm of expected dengue cases in week *t*, *α* is the model intercept; *T*_*t,l*_ and *R*_*t,l*_ are the matrices obtained by applying the DLNM to temperature and rainfall; *β*1 and *β*2 are the coefficients of temperature and rainfall matrices, *l* is the lag in months; *L* is the maximum lag; *s*(week, *λ*) is the natural cubic spline smoothing function of the calendar week. The model was adjusted by using a natural cubic spline for temperature with a maximum lag of 4–30 weeks, whereas base B-spline function was used for rainfall with a maximum lag of 25 weeks. We used the R software (version 3.3.3) with the ‘dlnm’ package to carry out our analysis [38].

Pearson cross-correlation coefficient test was used to investigate the lagged relationship between monthly dengue cases, ENSO and IOD data. The level of statistical significance was considered to be 0.05. The correlation analysis was calculated by using the SYSTAT statistical software (version 13).

## Results

The weekly dengue cases, temperature and rainfall from 2010 to 2017 are shown in Fig. 1. Between January 2010 and December 2017, a total of 174 912 dengue cases were reported by IDSP in India. There was an average of about 420 weekly dengue cases and 21 864 annual dengue cases. The highest number of cases (15 619) was reported during the 45th week of 2015 (e.g. first week of November). The weekly mean temperature and average rainfall were 23 °C and 23 mm, respectively. Descriptive statistics of all dependent and independent variables are shown in Table 1. The majority of dengue cases (72.47%) occurred from July to October during the monsoon period (Fig. 1). During this period, the median maximum and minimum temperatures were 28 and 21 °C, respectively. It reveals that the weekly time-series dengue cases and climatic factors indicate a strong seasonal pattern. The seasonal peak of dengue cases varies from year to year, although most of the cases tend to occur during the Indian monsoon (Fig. 1). Dengue cases increased tremendously from 2012 to 2016; 2015 and 2016 were considered as major dengue epidemics in India. There is no synchronous relationship between weekly dengue cases and climatic factors but a delayed lag effect was detected.

**Fig. 2.**
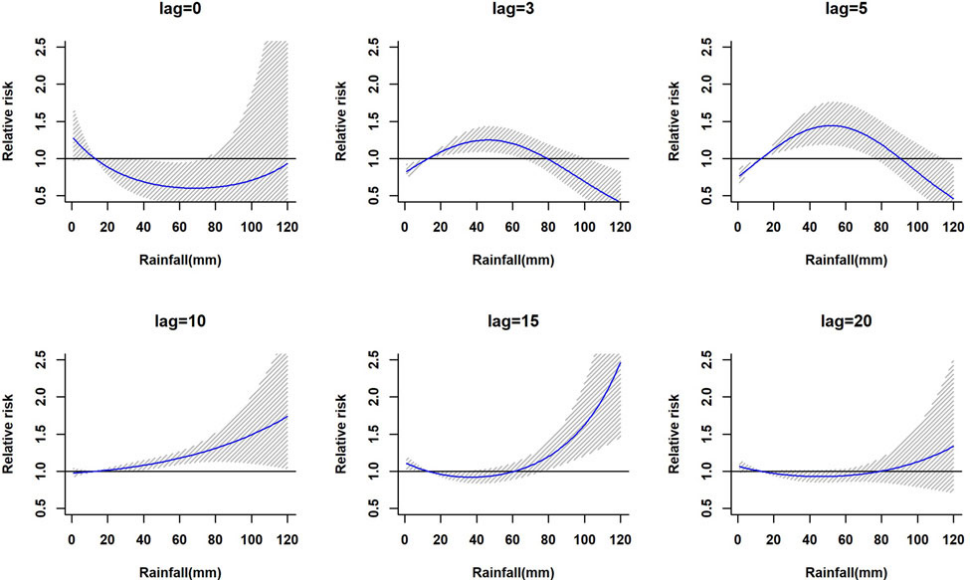
The estimation of relative risk posed by rainfall at different time lags (in weeks). The solid blue line is the estimated non-linear curve; the shaded region indicates its 95% confidence interval.

**Fig. 3.**
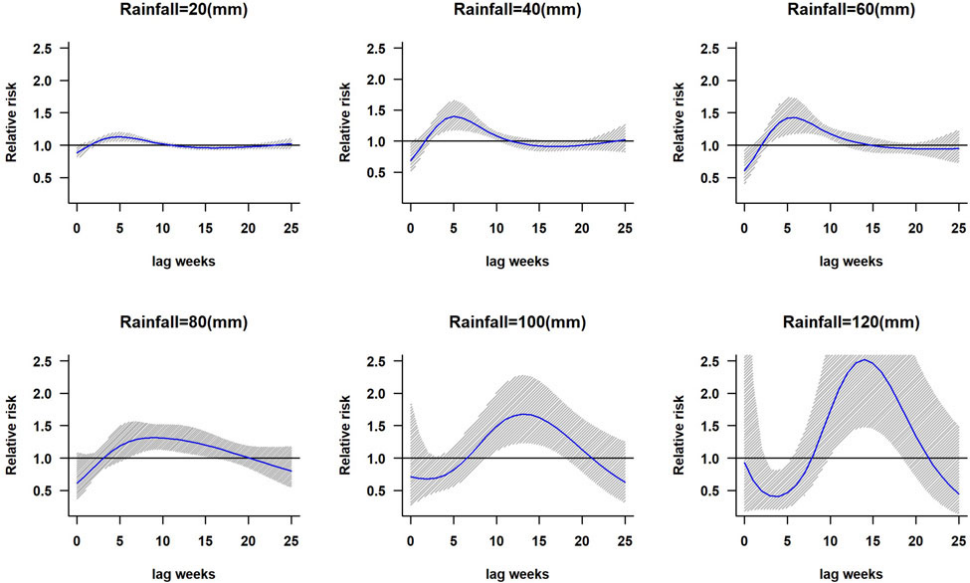
The relative risk of dengue at different rainfall ranges. The solid blue line is the estimated non-linear curve; the shaded region indicates its 95% confidence interval.

The lagged relationship between weekly cumulative rainfall and dengue cases in three-dimensional pattern is shown in Fig. S1. It reveals a non-linear relationship between rainfall and dengue cases with delayed lag effect. The RR is minimal for low rainfall period and it gradually increases along with increases in rainfall. The peak risk (RR > 2.5) was observed between 100 and 120 mm rainfall with 9–20 weeks lag, which decreased slowly during the following week. Figure 2 shows cross-sections of the two-dimensional surface shown in Fig. S1 for fixed time lags at 3, 5, 10, 15 and 20 weeks lag periods and for specific rainfall values, e.g. for 20, 40, 60, 80, 100 and 120 mm. The exposure–response analyses indicate that moderate dengue risk is observed between 40 and 60 mm rainfall with a 5-week lag period (Fig. 3). Rainfall ranging between 80 and 120 mm was associated with a higher RR and longer lag periods were observed (Fig. 3).

**Fig. 4.**
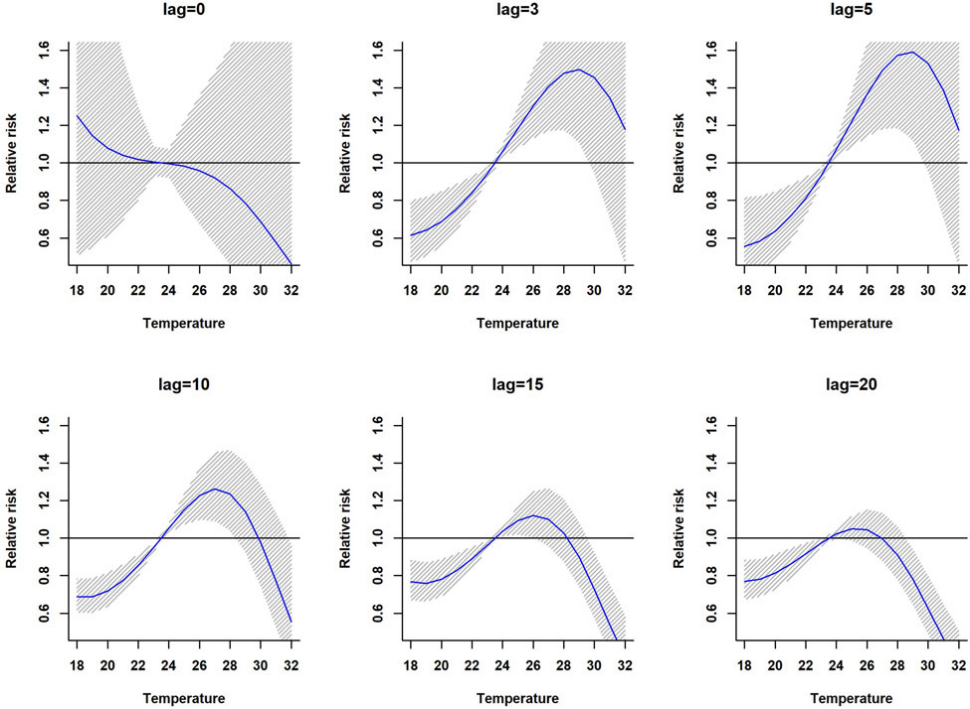
Relative risk by mean temperature at specific lags. The solid red line is the estimated linear curve, with shaded region indicating its 95% confidence interval.

**Fig. 5.**
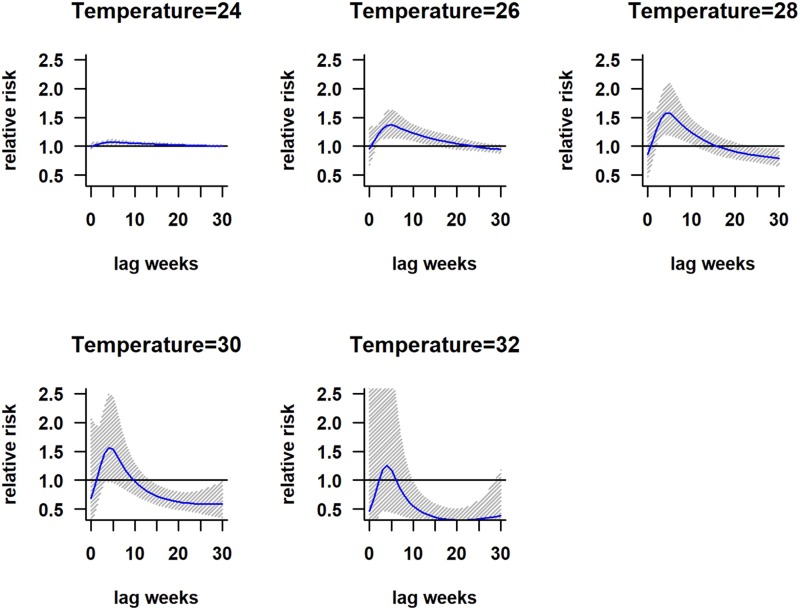
Relative risk by lag at different mean temperatures. The solid red line is the estimated linear curve, with shaded region indicating its 95% confidence interval.

**Fig. 6.**
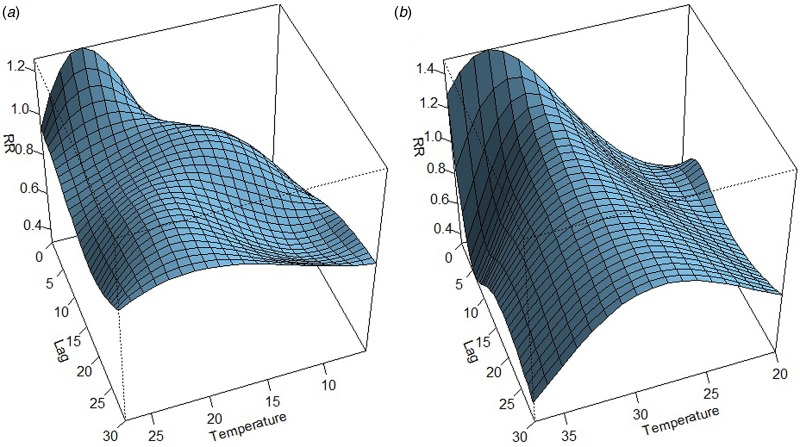
The three-dimensional plot shows the association between weekly. (a) Minimum temperature. (b) Maximum temperature and relative risk of dengue at different lags.

The association between mean temperature and dengue cases with a 30-week lag is shown in Fig. S2. The three-dimensional relationship between mean temperature and dengue cases shows a non-linear relationship and the RR increases with increasing temperature (Fig. S2). The largest RR (1.8) is shown between 28 and 30 °C with a 3–8 weeks lag. Thereafter the risk gradually decreases with increasing temperature (>30 °C). A reversed U-shaped lag responsive curve relationship was observed between dengue and mean temperature at different lag periods (Fig. 4). The exposure–response analyses (Fig. 4) highlight that the RR by temperature at specific lags (3, 5, 10, 15 and 20 weeks) and by lag at specific temperature (24, 26, 28, 30 and 32 °C) was observed. It was found that, the risk of disease transmission increases with increasing mean temperatures (*T*) but high risk of disease transmission was further observed at optimum mean temperature range (28–30 °C) and the risk of disease transmission decreases for *T* < 24 °C and *T* > 32 °C (Fig. 5). The associations between minimum and maximum temperature and dengue RR are presented as three-dimensional graphs in Fig. 6. The dengue RR is higher (>1.2) when temperature leads RR by 1 week and when minimum temperature ranges between 22 and 25 °C. RR > 1.4 is shown with 1 week lag when maximum temperature lies between 32 and 35 °C.

### Cross-correlation

**Fig. 7.**
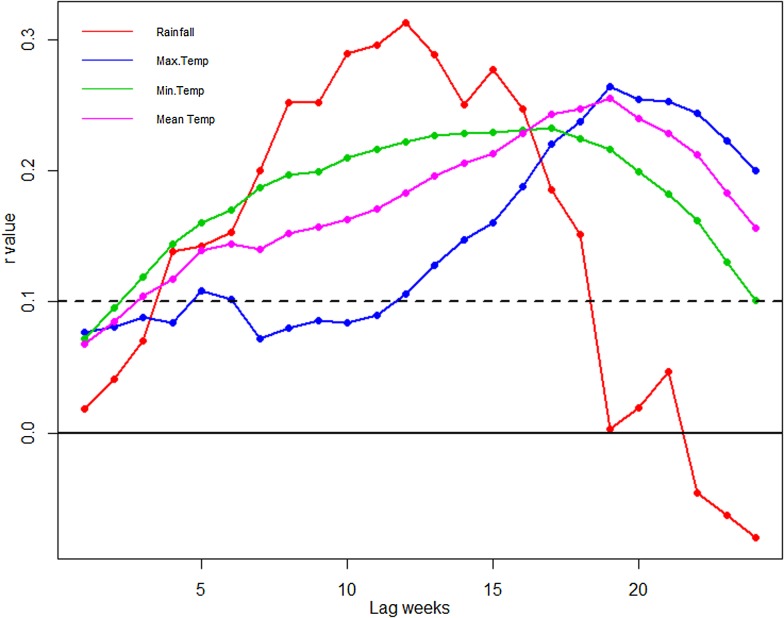
Cross-correlation of dengue cases and climatic variable at 0–25 weeks time lag. The dotted line stands for the significant correlation coefficients with *P* < 0.05.

Temperature and rainfall exhibited significant correlations with dengue cases at different time lags (Table S1). The largest correlation (*r* = 0.32) is shown between rainfall and dengue at 12-week lag (Fig. 7). The positive correlation coefficient is observed between maximum temperature and dengue cases. Mean temperature also shows positive correlation with dengue cases at a 19-week lag period. The correlation between minimum temperature and dengue is significant but the correlation values are quite small.

### Effect of ENSO and IOD on dengue

**Fig. 8.**
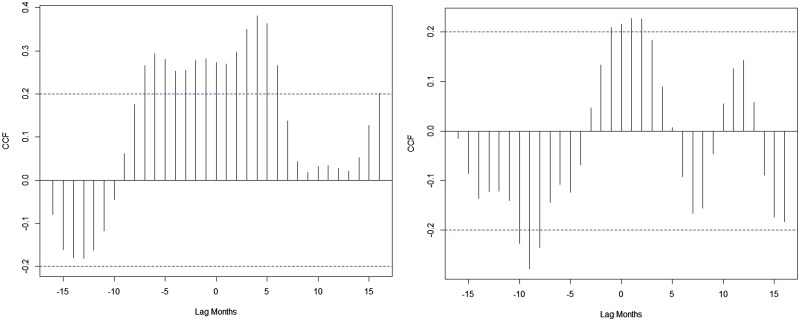
Cross-correlation between NIÑO3.4, DMI indices and dengue cases.

A weak synchronous correlation was observed between Nino3.4 (*r* = 0.3), DMI (*r* = 0.2) and dengue cases from 2010 to 2017. However, the largest El Niño events and the largest positive phases of the IOD coincide with the largest number of dengue cases reported in India in 2015 and 2016 (Fig. 1). Good correlation coefficients are shown between Nino3.4 (*r* = 0.5), DMI (*r* = 0.6) and dengue cases during strong El Niño period (in 2015), whereas negative correlation (*r* = −0.6) between dengue cases and Nino3.4 and DMI indices are shown for 2016. Figure 8 depicts cross-correlations between Nino3.4 and DMI indices with dengue cases. Monthly Nino3.4 and DMI indices were significantly associated with the number of dengue cases at different time lags. Nino3.4 shows the largest significant correlation coefficient when the Nino3.4 index lead dengue cases by 3–6 months. Similarly, the highest significant correlation between the DMI index and dengue cases was found for a time lag ranging between 0 and 2 months (Table S2).

## Discussion

Many studies have shown that temperature and rainfall are important drivers associated with the emergence of dengue. In the present study, a DLNM was utilised to understand the short-term association between climate variables and dengue cases for different time lags. The results show that minimum temperature (26 °C), maximum temperature (32 °C) with 0–5 weeks lag and rainfall (60 mm) with a lag of 8–12 weeks are the most significant variables associated with an increase in the RR of dengue.

The non-linear relationship between rainfall and dengue is related to rainfall effects on the adult female mosquito lifecycle. Rainfall provides breeding habitats and opportunities for the proliferation of vectors in the environment. An increase in the amount of rainfall leads to more potential breeding sites, which, in turn, lead to an increase in the number of mosquitoes hatching. However, high rainfall may washout mosquito breeding sites thus having a negative effect on mosquito density [15]. This is consistent with our findings, the RR of dengue gradually increases as cumulative weekly rainfall increases from 40 to 60 mm, then it decreases when rainfall exceeds 80 mm. Similar results were observed in Brazil, namely that the risk of dengue infection increases during the rainy season when vector infestation reaches its peak [39]. Excepting rainfall intensity and magnitude, other man-made factors such as human activities, usage of water, storage patterns and drainage systems can create artificial breeding habitats for dengue vectors. In Southeast Asian countries, residents generally grow potted plants/flowers indoors and they tend to decorate roofs with hanging gardens. These man-made containers, if not emptied or cleaned frequently, can act as a breeding source for vectors well adapted to the urban environment. This can potentially further increase the risk of dengue transmission [40, 41]. In India, residents generally store water in different containers inside the house (especially in urban settings) and this water acts as a breeding source for *Aedes* vectors, thus further increasing the risk of dengue transmission [9]. In India, the south-west monsoon brings enough rainfall to sustain vector breeding sites [42, 43]. Our findings show that the seasonality of the mosquito population is mainly driven by rainfall and ambient temperature conditions.

Temperature is one of the most important climatic factors, which influences the life cycle of the mosquito and pathogen development inside the vector. Temperature influences the length of gonotrophic cycle, larval development and growth rate of *Aedes* mosquitoes [44]. High temperatures are also associated with an increase in dengue incidence due to faster viral replication rates, shortened extrinsic incubation periods, increased blood-feeding behaviour, low mortality rates and high mosquito biting rates, leading to increased vectorial capacity. All these factors play a key role in disease transmission [15, 45]. Previous studies have investigated the effect of temperature on the burden of dengue fever [46]; weekly minimum temperature was also strongly associated with vector development [47, 48]. In this study, a positive association was observed between temperature and dengue cases for temperatures ranging between 24 and 30 °C at a lag of 0–4 weeks. Similar temperature ranges were also observed in China and Mexico [49, 50].

Studies found that water-borne disease and VBD outbreaks coincide with ENSO and IOD events [51, 52]. The present study found a weak synchronous correlation between ENSO (*r* = 0.3), IOD (*r* = 0.21) and dengue cases. In 2015–2016, a large number of dengue cases were observed during one of the largest El Niño events, a large positive phase of the IOD along with high rainfall. El Niño events are associated with a warming signal over the whole Tropics, warming temperature brought changes in the atmospheric circulation over the Indian Ocean which favours large rainfall during the monsoon period [31]. A significant positive relationship was observed between ENSO and monthly dengue incidence in Pacific Island nations [53]. Similarly, countries like Thailand, Mexico and Bangladesh have shown a positive association between ENSO, IOD and dengue cases [27, 54, 55].

The lagged effect of ENSO and IOD on the risk of dengue fever transmission is very important. The observed lag effects are biologically plausible, and they are consistent with former findings [52, 56, 57]. A study compiling all historical dengue outbreaks in India showed that most outbreaks in India are highly seasonal (occurring during the monsoon period) [9]. This study suggests that rainfall occurring at the end of the dry season enhances the risk of epidemics during the following monsoon season. Conversely, during pronounced drought conditions, water containers help to maintain the *Aedes* vector population around man-made water reservoirs in households. As a consequence, the pathogen remains during the dry season, and this can lead to an epidemic when the wet season returns. Non-climatic factors such as socio-ecological changes, viral serotypes, immunological factors, mosquito control approaches and population movement are important drivers for the spatial and temporal dynamics of dengue fever transmission [58, 59].

Earlier researchers found that dengue and its vectors are adapted to an urban setting, but in India, it has also spread to rural regions [1, 60, 61]. In recent years, the number of dengue cases has increased dramatically in India; hence it is crucial to develop a seasonal forecasting model to predict dengue prevalence for the next season. Our study has provided some basic information on temperature and rainfall threshold levels which might help to build a dengue early warning system to inform decision-making activities such as when to initiate preventive measures to reduce dengue mortality and morbidity.

This study has some limitations. First, the study duration (2010–2017) and sample size is relatively small. Our study highlights the granular association between climate and dengue at country scale. Similar modelling exercise should be carried out at city or state level for effective management and control of the disease. As India has different climatic zones, future studies should focus on the development of forecasting models by climatic zone. Other important parameters, including socioeconomic and demographic factors such as population density, migration, vector density, virus serotype and immunity of the population should be included in future risk assessment studies to further understand this complex and fast-growing disease.

## Conclusion

In conclusion, our findings revealed a non-linear relationship between climatic factors and dengue burden in India. The study shows that dengue is temperature-dependent and with increasing temperature the dengue cases increase above 24 °C. The estimated lagged effects are in accordance with the time required for the development of the *Aedes* vectors, for the extrinsic and intrinsic incubation periods of virus as well as the onset of clinical symptoms of dengue. The study also provides information for better understanding the effect of climate variables on dengue and can adapt a policy for control and preventive measures well in advance.

## Supplementary material

For supplementary material accompanying this paper visit https://doi.org/10.1017/S0950268819000608. click here to view supplementary material
