# Supplementary material for: Lag effect of climatic variables on dengue burden in India
Source: Epidemiol Infect. 2019 Apr 3;147:e170. doi: 10.1017/S0950268819000608 (PMC6518529; doi:10.1017/S0950268819000608)
Supplement: Supplementary file 1 [file S0950268819000608sup001.zip › Supporting_tables_S1-S2.docx]

**Supporting Tables:**

| **Time lag weeks** | **Temperature** | | | **Rainfall** |
| --- | --- | --- | --- | --- |
|  | **Maximum** | **Minimum** | **Mean** |  |
| 1 | 0.077 | 0.072 | 0.068 | 0.018 |
| 2 | 0.081 | 0.095 | 0.085 | 0.041 |
| 3 | 0.088 | 0.119 | 0.104 | 0.07 |
| 4 | 0.084 | 0.144 | 0.117 | 0.138 |
| 5 | 0.108 | 0.16 | 0.139 | 0.142 |
| 6 | 0.102 | 0.17 | 0.144 | 0.153 |
| 7 | 0.072 | 0.187 | 0.14 | 0.2 |
| 8 | 0.08 | 0.197 | 0.152 | 0.252 |
| 9 | 0.086 | 0.199 | 0.157 | 0.252 |
| 10 | 0.084 | 0.21 | 0.163 | 0.289 |
| 11 | 0.09 | 0.216 | 0.171 | 0.296 |
| 12 | 0.106 | 0.222 | 0.183 | 0.313 |
| 13 | 0.128 | 0.227 | 0.196 | 0.288 |
| 14 | 0.147 | 0.228 | 0.206 | 0.25 |
| 15 | 0.16 | 0.229 | 0.213 | 0.277 |
| 16 | 0.188 | 0.231 | 0.228 | 0.247 |
| 17 | 0.22 | 0.232 | 0.243 | 0.185 |
| 18 | 0.237 | 0.224 | 0.247 | 0.151 |
| 19 | 0.264 | 0.216 | 0.255 | 0.003 |
| 20 | 0.254 | 0.199 | 0.24 | 0.019 |
| 21 | 0.253 | 0.182 | 0.228 | 0.047 |
| 22 | 0.244 | 0.162 | 0.212 | -0.046 |
| 23 | 0.223 | 0.13 | 0.183 | -0.063 |
| 24 | 0.2 | 0.101 | 0.156 | -0.08 |

Table S1: Cross-correlation coefficients between climate variables and dengue incidence.

| **Time lag months** | **NINO3.4** | **DMI** |
| --- | --- | --- |
| 0 | 0.28 | 0.23 |
| 1 | 0.27 | 0.24 |
| 2 | 0.29 | 0.23 |
| 3 | 0.35 | 0.18 |
| 4 | 0.38 | 0.09 |
| 5 | 0.37 | 0.02 |
| 6 | 0.27 | -0.09 |
| 7 | 0.14 | -0.18 |
| 8 | 0.04 | -0.16 |
| 9 | 0.02 | -0.04 |
| 10 | 0.04 | 0.08 |
| 11 | 0.04 | 0.14 |
| 12 | 0.03 | 0.14 |

Table S2: Cross-correlation coefficients between global climate variables and dengue incidence.
