# Supplementary figures and images for: Lag effect of climatic variables on dengue burden in India
Source: Epidemiol Infect. 2019 Apr 3;147:e170. doi: 10.1017/S0950268819000608 (PMC6518529; doi:10.1017/S0950268819000608)

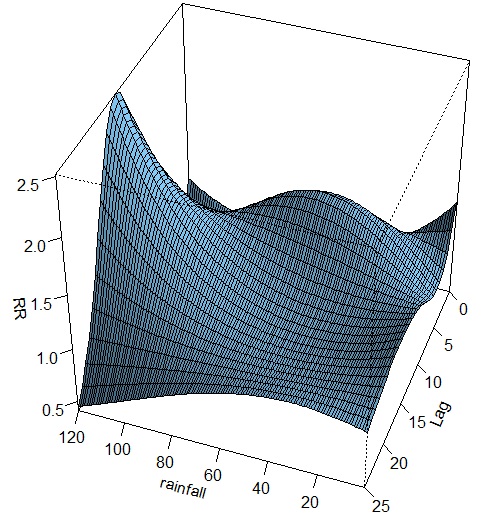

Supplement: Supplementary file 1 [file S0950268819000608sup001.zip › Figure-S1.jpg]

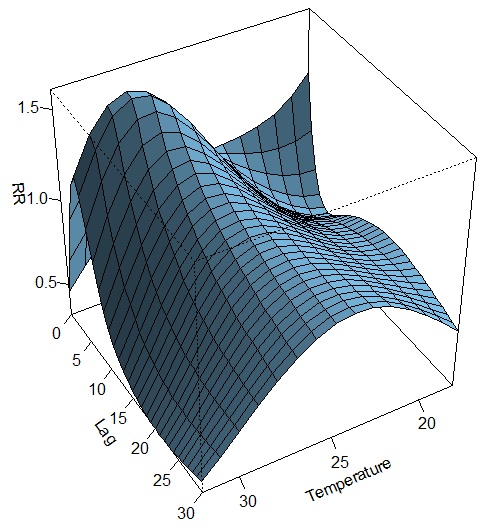

Supplement: Supplementary file 1 [file S0950268819000608sup001.zip › Figure-S2.jpg]
